# Molecular swarm robots: recent progress and future challenges

**DOI:** 10.1080/14686996.2020.1761761

**Published:** 2020-06-16

**Authors:** Arif Md. Rashedul Kabir, Daisuke Inoue, Akira Kakugo

**Affiliations:** a Faculty of Science, Hokkaido University, Sapporo, Japan; b Faculty of Design, Department of Human Science, Kyushu University, Fukuoka, Japan; c Graduate School of Chemical Sciences and Engineering, Hokkaido University, Sapporo, Japan

**Keywords:** Molecular robot, molecular engine, swarming, biomolecular motor, synthetic motor, DNA, sensor, actuator, processor, photoresponsive molecules, 101 Self-assembly / Self-organized materials, 208 Sensors and actuators

## Abstract

Recent advancements in molecular robotics have been greatly contributed by the progress in various fields of science and technology, particularly in supramolecular chemistry, bio- and nanotechnology, and informatics. Yet one of the biggest challenges in molecular robotics has been controlling a large number of robots at a time and employing the robots for any specific task as flocks in order to harness emergent functions. Swarming of molecular robots has emerged as a new paradigm with potentials to overcome this hurdle in molecular robotics. In this review article, we comprehensively discuss the latest developments in swarm molecular robotics, particularly emphasizing the effective utilization of bio- and nanotechnology in swarming of molecular robots. Importance of tuning the mutual interaction among the molecular robots in regulation of their swarming is introduced. Successful utilization of DNA, photoresponsive molecules, and natural molecular machines in swarming of molecular robots to provide them with processing, sensing, and actuating ability is highlighted. The potentials of molecular swarm robots for practical applications by means of their ability to participate in logical operations and molecular computations are also discussed. Prospects of the molecular swarm robots in utilizing the emergent functions through swarming are also emphasized together with their future perspectives.

## GRAPHICAL ABSTRACT

**Figure:**
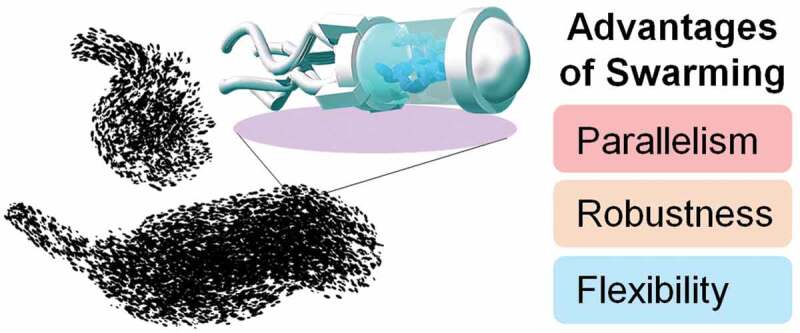

## 1. Introduction

**Figure 1.**
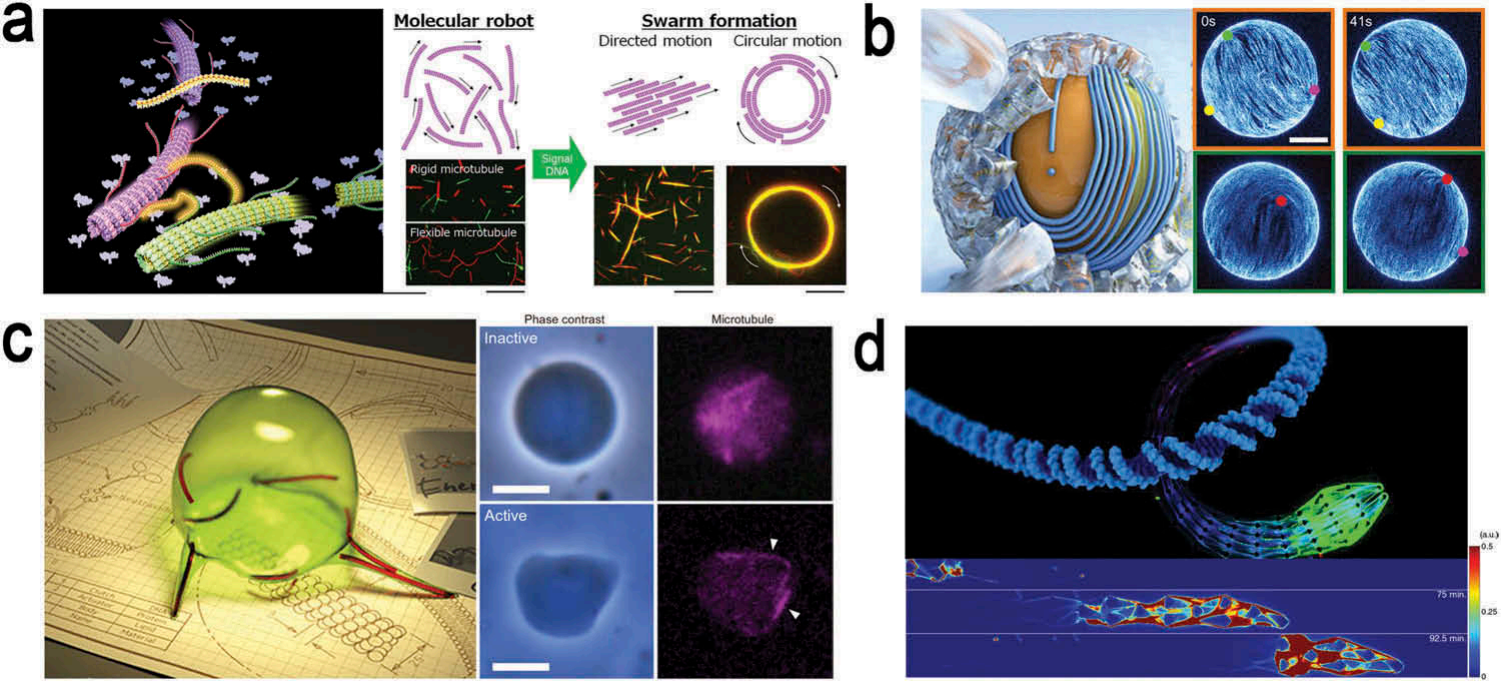
Recent advances in molecular robotics in which DNA nanotechnology played a crucial role. (a) Conjugation of DNA to microtubules to demonstrate swarming of microtubules like robots when they were driven by kinesin motor protein; reproduced with permission from [34]; (b) Fabrication of a self-propelled particle by encapsulating a film of microtubules and kinesins inside a lipid vesicle; Image by Christoph Hohmann (LMU) & Etienne Loiseau (TUM); reproduced with permission from [35]; (c) Biomolecular motor microtubule, kinesin, and DNA clutch were encapsulated inside a vesicle which exhibited amoeboid motion [36]; Illustration copyright: Sho Aradachi; reproduced with permission from [36]; (d) Artificial metabolism driven dynamic locomotion of DNA-based materials; reproduced with permission from [37].

Over the last decade, we have witnessed enormous progress in the development of artificial molecular machines, as exemplified by the 2016 Nobel Prize in Chemistry [1,2]. An ability to manipulate molecules has greatly facilitated the recent development of artificial molecular machines which have been proved promising in performing specific tasks. With such progress, a new paradigm towards molecular robotics has emerged through the fusion of various fields, thanks to the latest innovations in supramolecular chemistry, nanotechnology, chemical engineering, biomolecular engineering, etc. [3–13]. The artificial molecular machines have been proved effective in accomplishing various tasks like molecular robots, i.e. a device or a system which can perform tasks autonomously by assessing its surrounding based on a program or information provided. Molecular robots have been reported to be useful in oligomer synthesis [14,15], switching of product chirality [16,17], mechanically twisting molecules [18], molecular transportation [19] and moving a substrate between different activating sites to achieve different product outcomes from chemical synthesis [20]. In the latter case, the molecular robots possess programmability for stereoselective conversion of reactants into products in chemical reactions. Considerable efforts have also been devoted to fabricating nanocar or nanotruck with controlled motion from fullerene [21–23]. Swimming molecular robots energized by external magnetic fields have attracted attention in recent years that exhibited a variety of intriguing dynamic behaviors [24]. Apart from the many attempts based on synthetic or supramolecular chemistry, DNA nanotechnology and bioengineering also came up with great promises in the advancements of molecular robots [25] (Figure 1). DNA-based well-designed and robust molecular machines like DNA walkers [26], nanomotors [27], switches [28], nanorobotic arm [29], etc. have been fabricated that can perform specific functions at nanoscale. The DNA nanorobotic arm was synthesized from a six-helix DNA bundle connected to a DNA origami plate via flexible single-stranded scaffold crossovers [29]. The arm could be driven by externally applied electrical fields and can be used for transport of molecules or nanoparticles, which would be useful for the control of photonic and plasmonic processes.

Living organisms perform tasks which are essential for life by using the molecular machines already available in nature. Some of the examples include moving large cargoes in cells using biomolecular motors [30], converting chemical energy from one form to another using ATP synthase [31], replicating biopolymers using DNA synthase [32], performing complex chemical synthesis using ribosome [33], etc. Therefore, alongside the synthesis of molecular machineries, sophisticated molecular machines available in nature have also attracted much attention. Employing the natural machineries to work in synthetic environments has appeared as another promising approach to fabricate biomimetic molecular robots. As an example, micrometer-sized molecular robots, capable of programmed swarming, have been developed from a biomolecular motor system microtubule-kinesin [34]. An active nematic film of microtubules and biological molecular motors encapsulated within a lipid vesicle was reported that worked as an autonomous particle [35]. In another work, combination of vesicles, biomolecular motor proteins, and DNA clutch expressed amoeba-like motion [36]. An artificial metabolic system was successfully used to drive dynamic DNA materials with emergent locomotion as an artificial biological system [37].

**Figure 2.**
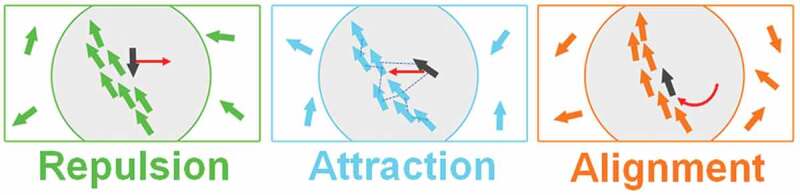
In the flocking of self-propelled objects such as living organisms, three modes of interactions among the dynamic units are important: repulsion, attraction, and alignment. Repulsion helps maintain an appropriate distance to prevent collision or crowding among the objects. Attraction permits formation of assembly of the objects. Alignment facilitates coordinated motion of the dynamic objects.

In these developments very small size of the molecular robots has been a big advantage since it enables creating a large number of robots and managing them to perform tasks coherently. Allowing small individuals to interact with each other and produce a more complex, collective task, has been motivated by collective behavior of living organisms [38,39]. Local interactions among the organisms play the key role in the emergence of such flocks. Three types of interactions such as attraction, repulsion, and alignment (Figure 2) are worth to consider in the flocking exhibited by living organisms such as birds, fish, cells, and bacteria [40,41]. Living organisms can process information received through interactions with nearby individuals and cooperatively change their organization, e.g. position, shape, or size of the groups, even in the absence of any leader. Flocking facilitates several emergent functions to the organisms such as parallelism, robustness, and flexibility which are unachievable by a single entity [39]. Parallelism enables sharing of tasks by creating groups, robustness helps to ensure that tasks are executed properly, and flexibility permits the flocks to respond instantaneously to their environments. With a view to demonstrate swarming of molecular robots by controlling their mutual interactions, in order to exploit these advantages, enormous efforts have been devoted to construct mechanical or chemically fueled self-propelled systems [42–47]. Swarming of chemically fueled self-propelled systems was regulated by manipulating their mutual interactions through chemical signaling, electrostatic forces, magnetic forces, or other forces, etc. [45,46,48–53]. Scalability and population of the robots have been two major obstacles in demonstrating flocking of molecular robots. The size of a robot, made from hard materials and capable of swarming, has been limited to centimeter scale and the maximum number of robots that can be employed in flocking was one thousand [54]. Therefore, to make any swarming system more flexible and expandable, reducing the size of individual units further and increasing the number of individuals is crucial. In order to address these challenges, it was indispensable to have further control on the ‘size’ and ‘number’ of individual robots that is inevitable for their applications in engineered systems. Recent developments in molecular robotics ensure two major advantages in swarming of the robots: since molecular robots are fabricated from molecular parts, their size is limited within nano-/microscale [34,36,37]. This in turn further facilitates an opportunity to employ a large number of robots to work in a concerted fashion which consequently allowed mimicking the autonomy and intelligence exhibited by living organisms. Therefore, scalability and tunability of population of molecular robots have been useful in overcoming the limitations of the conventional robots prepared from hard materials. More importantly, in the above studies that demonstrated swarming of self-propelled systems, the basic features of robots, such as sensor, actuator, and processors were unavailable. Therefore, the imminent challenge has been to introduce sensing, actuating, and processing ability to self-propelled systems. In the below sections we discuss how the recent efforts have been successful in overcoming most of these barriers and demonstrated swarming of the molecular robots that possess sensing, actuating, and processing ability.

### 2.1. What is a molecular robot?

**Figure 3.**
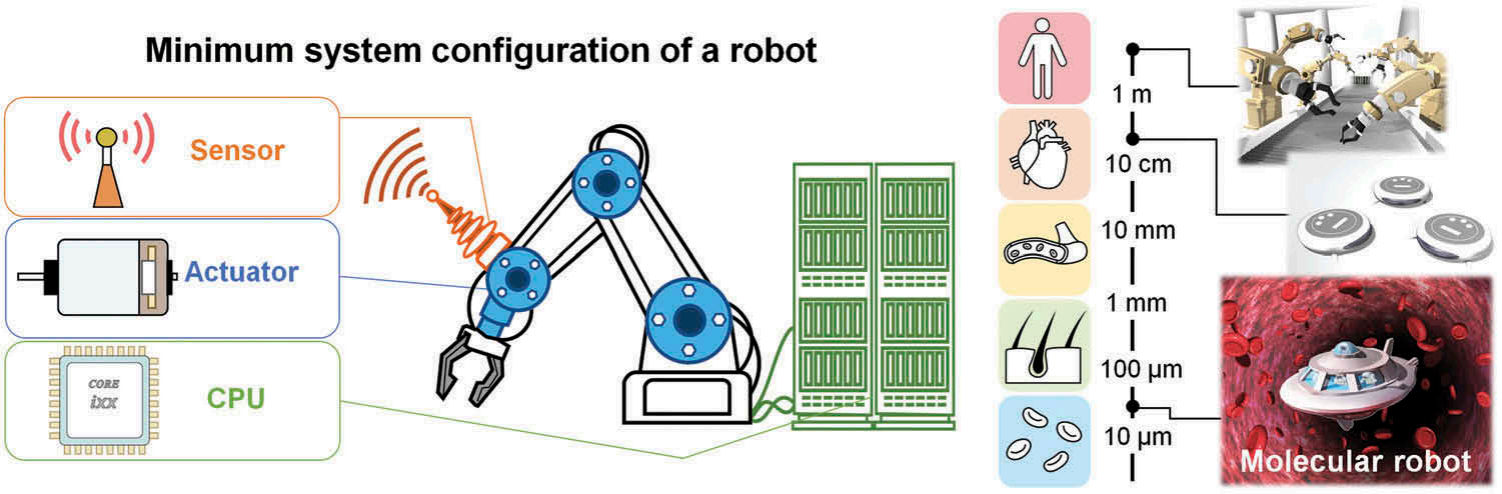
Schematic diagrams show three basic components of a robot, namely, sensor, actuator, processor (left); comparison of robots size of which ranges from meter to micrometer scale. The conventional robots are of micrometer scale whereas molecular robots are of micrometer scale (right).

A molecular robot can be best described as an integrated system formed through the combination of different molecular parts or devices that may work as processor or logic gates, actuator, and sensors [55] (Figure 3). A molecular robot is expected to be autonomous in receiving information from its surrounding and making decision through its own ability of molecular computation. As molecular sensors, various DNA/RNA-based nanostructures have been developed with a view to sense variety of signals and convert the information to output signals for other parts of the robots [56–65]. Photo-responsive molecules provide sensing ability to the robots [57,66]. As the information processor DNA-based devices, such as seesaw gates are promising [67]. Manipulation of DNA using photochemical technology has appeared a promising tool to construct novel devices for molecular computing [68]. Biomolecular motor proteins such as actin-myosin or microtubule-kinesin/dynein have been the best choices as actuators for molecular robots. Biomolecular motors can convert chemical energy into mechanical work with a remarkably high efficiency and hence have been promising as the actuator to drive synthetic systems [69–73]. Based on these ideas, recent progresses in the molecular robots enable precise designing of complex structures which may find a wide range of applications such as sensing, sequential signaling, adaptive actuating, etc. [20,34,74–76]. However, controlling the scalability and number of units of molecular robots in an ensemble remained challenging, and following initiatives were undertaken to overcome these limitations.

### 2.2. Design of molecular robots

Three basic components are essential for constructing molecular robots: actuator, processor, and sensor (Figure 3). Actuator serves as the power generator for the molecular robots. Processor works as a mechanism for processing information by the robots. Sensors are required to detect information by the robots from their surroundings. Recent advances in different branches of chemistry, e.g. polymer chemistry, organic chemistry, supramolecular chemistry, etc., biotechnology, e.g. genetic and protein engineering, etc., and nanotechnology have collectively led to the implementation of the idea of molecular robots. By integrating these molecular elements into a microscopic system in a bottom-up manner, a molecular robot was developed [34]. This molecular robot can exhibit self-propelling behavior both in a solitary state and in groups, which renders them suitable for demonstrating swarming. Among the many functional molecular parts, the biomolecular motors have been selected as the actuator for molecular robots. DNA has been used as the information processor, and photosensitive molecules such as azobenzene played the role of a sensor for the molecular robots.

### 2.3. Characteristics of the three elements constituting molecular robots

Biomolecular motor, the power source of a molecular robot, is the smallest natural molecular machine that can perform various tasks in cells by consuming chemical energy [77]. By virtue of the recent advancements in biotechnology, the biomolecular motors can be reconstructed nowadays. Biomolecular motors have an outstanding ability to convert chemical energy into mechanical work with relatively high energy conversion efficiency and specific power which compared to electromagnetic motors. For this reason, research and development of nanodevices and bioactuators using biomolecular motors as power sources have been actively conducted in recent years [71]. On the other hand, DNA is a storage medium for genetic information having high molecular recognition ability depending on base sequence information. The chemical synthesis of DNA has facilitated their applications to various fields as complex nanostructures (DNA origami), digital data recorder, and DNA computers that can solve mathematical problems [78,79]. The logical operation of DNA-based structures can be used as a processor that would be useful to control the molecular robots. Photosensitive molecules were used to regulate functions of molecular robots simply using light. An azobenzene derivative was incorporated into the DNA as a photosensitive molecule [34]. Under ultraviolet light irradiation, the computation of DNA could be turned off, which was again turned on by visible light irradiation. Thus, photosensitive molecules act as visual sensors for the molecular robots.

### 2.4. Integration of the three elements

**Figure 4.**
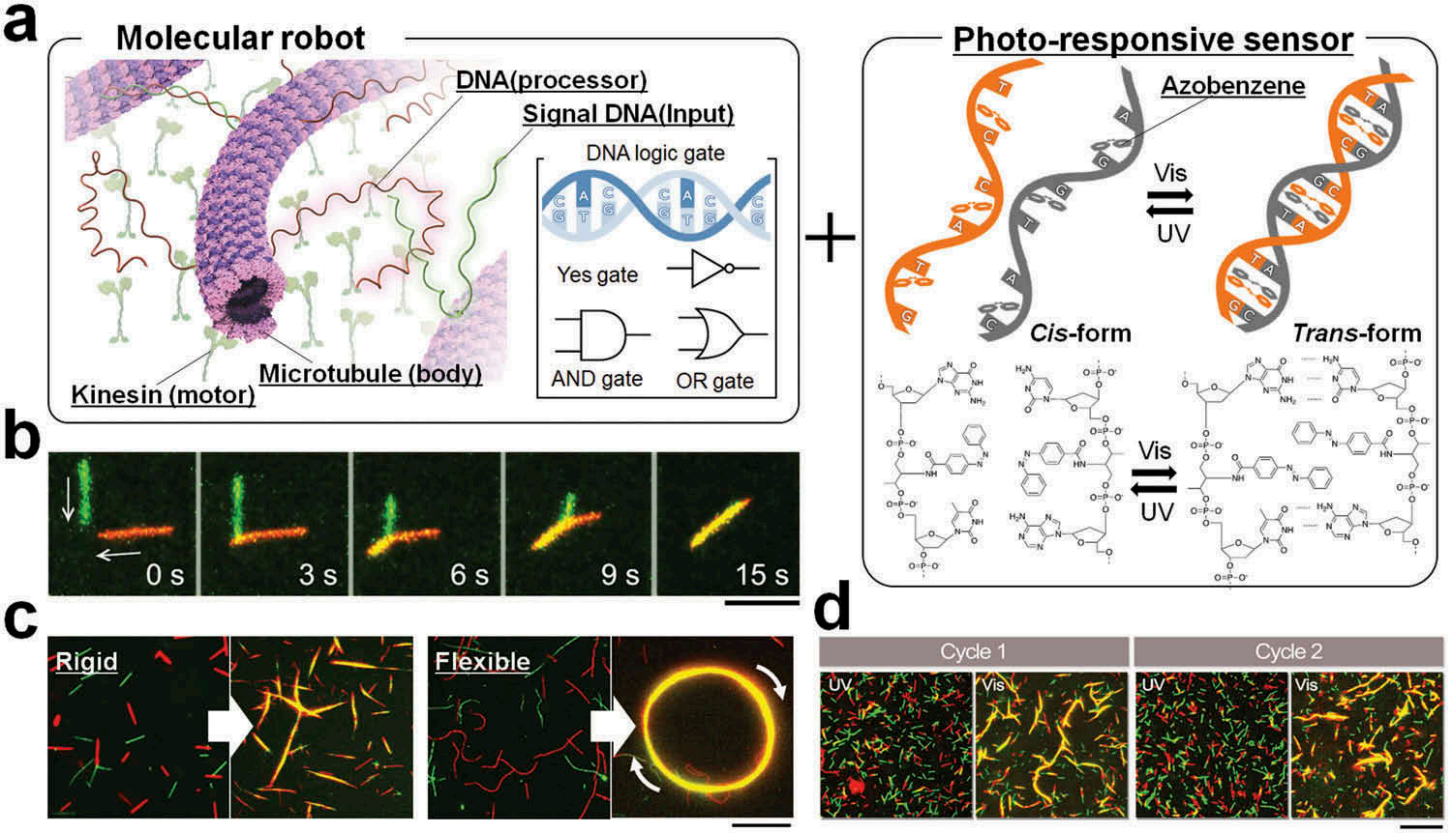
Preparation of molecular robots from microtubules and their swarming; reproduced with permission from [34]. (a) Microtubules are conjugated with single-strand DNA with complementary sequence. Azobenzene, a photoresponsive molecule, is inserted in the DNA to facilitate photoregulation of hybridization of the complementary sequences. (b) Microtubules, gliding on a kinesin-coated substrate, form swarm through self-organization due to hybridization of complementary DNA sequences. (c) Pattern of swarm can be tuned by simply changing the mechanical properties of microtubules. (d) Photo-regulated swarming of microtubules as robots. Scale bar: 20 µm.

Microtubule/kinesin, a cytoskeletal motor protein system, was used as the biomolecular motor for fabricating molecular robot. The basic unit of the molecular robot was prepared by using a chemical technique to conjugate microtubules with single-strand DNA (Figure 4). Furthermore, photo-responsiveness was incorporated by introducing an azobenzene derivative into the DNA. The activity of the prepared molecular robots was evaluated from their dynamics on a kinesin motor protein-coated substrate. The molecular robots exhibited gliding motion on the kinesins with an average velocity of ~600 nm/s, which is very similar to the velocity of microtubules without DNA conjugation [34]. Almost 85% of kinetic characteristics of microtubules were retained despite conjugation of DNA [34].

### 2.5. Demonstration of swarming by molecular robots

As already discussed, local interaction plays an important role in group formation by self-propelled objects. Swarming of molecular robots was realized by utilizing the molecular recognition ability of DNA in controlling local interactions between molecular robots [34]. Using an association DNA as an input signal, a large number of molecular robots gliding on a kinesin-coated substrate formed large swarm (Figure 4). The input DNA strand is designed such that it can mediate the interaction between motile molecular robots. In a swarm of molecular robots, all individuals moved in the same direction which is determined by the polarity of microtubules. DNA-based computation was used not only for swarm formation but also for dissociation of the swarms into single molecular robots. The input signal of dissociation DNA prompted the groups of molecular robots to separate into single robots through strand displacement reaction of the complementary DNA of neighbor robots.

### 2.6. Controlling morphology of swarming of molecular robots

The morphology of the swarms of molecular robots was also varied not only by local interactions introduced via DNA but also by tuning the length and rigidity of microtubules that were the basic units of molecular robots. For example, a molecular robot synthesized from microtubules having relatively high bending stiffness of 62 × 10^−24^ Nm^2^ [80] formed a linear bundle-shaped swarm and exhibited translational motion (Figure 4). On the other hand, when the stiffness of microtubules was reduced by changing their polymerization conditions, ring-shaped swarms were formed that exhibited rotational motion either clockwise or clockwise direction. The rigidity of microtubules can be controlled not only by changing their polymerization conditions but also using some stabilizers such as taxol or microtubule-associated proteins [80]. Changes in morphology of swarm robots are also found to affect the path-persistent length of the robots [34].

### 2.7. Logical operation of molecular robots

**Figure 5.**
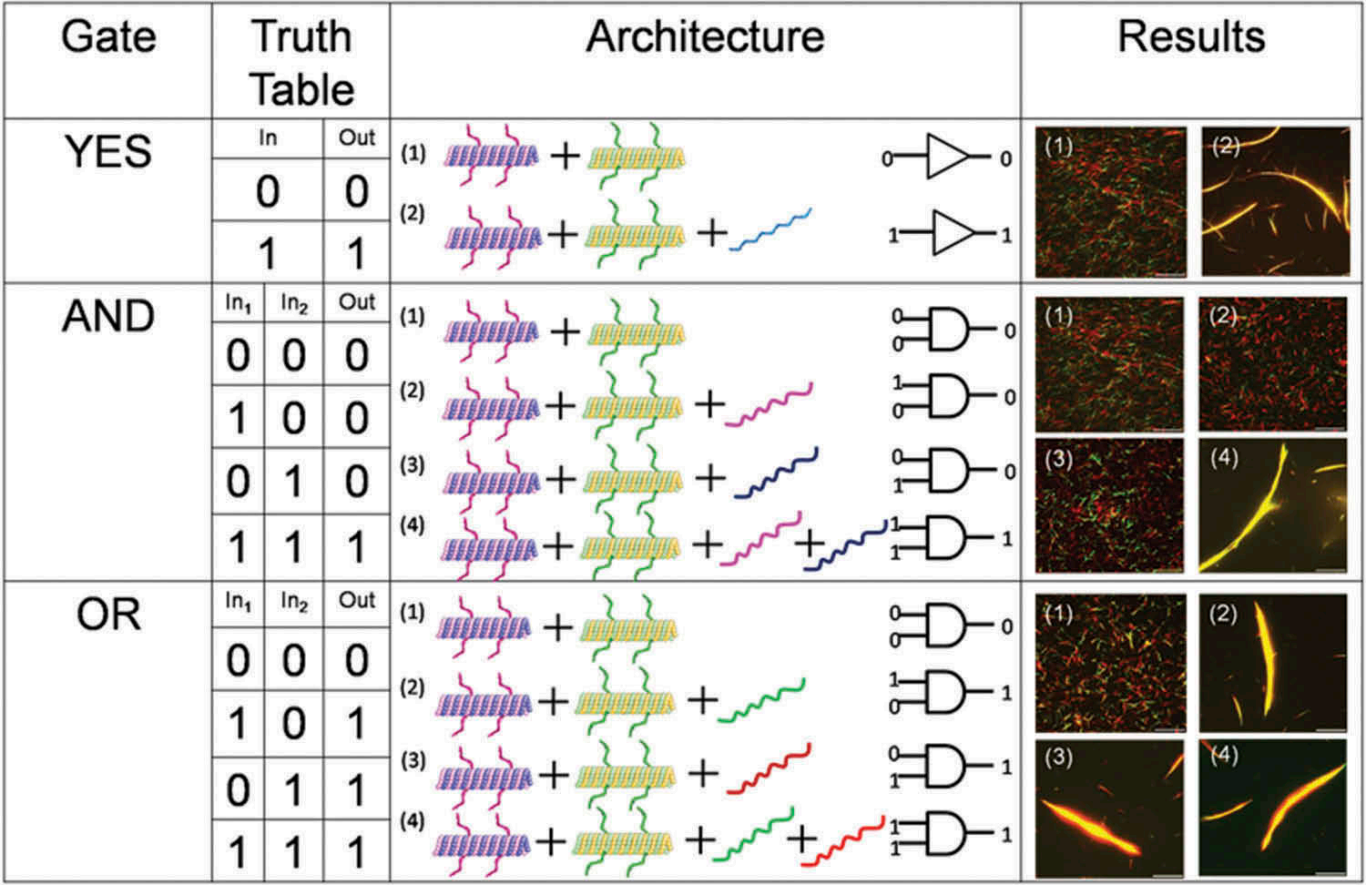
Design of logic gates constructed using molecular robots from microtubules; reproduced with permission from [34]. For the YES gate, a suitable DNA signal (DNA-1) was inputted into the system and swarming was obtained as the output signal (1 to 1). For the AND gate, DNA-2 and DNA-3 were necessary to be present to obtain swarming. For the OR gate, the presence of either DNA-1 or DNA-4 was enough to obtain swarming.

Over the last decade, extensive attempts have been undertaken in pursuit of more powerful computers. Looking beyond the currently used silicon chip, a promising alternative has been DNA which is able to perform more complex computing [81]. An ability to store incredible amount of data allows DNA to perform many calculations in parallel which has been a major advantage of DNA-based computing. Utilizing the ability of DNA to serves as a logic operator in molecular computing, different logic operations such as YES, AND, OR gate, etc., were demonstrated by the molecular robots [34]. In those logic operations swarming of molecular robots was the output that was regulated by suitable DNA signals as inputs (Figure 5). For example, a YES logic gate was realized by using an input DNA signal, presence of which facilitated swarming molecular robots (microtubules) that were already equipped with DNA signals complementary to the input DNA signal. The AND logic gate was demonstrated by designing two different input DNA signals, which were partially complementary to the two DNA signals carried by two groups of molecular robots. Swarming of the molecular robots was observed as the output only when both the input DNA signals were present. The OR logic gate was operated by simultaneous operation of two swarming groups. In each group, two types of molecular robots were equipped with two different DNA signals. The two types of robots exhibited swarming independently when another input DNA signal partially complementary to the DNA carried by the robots was available. Both the swarm groups were operated in a concerted fashion when both the input DNA signals were available in the same swarm system. In all the logical operations, the output can be confirmed visually from a change in the morphology of the swarm of the molecular robots or from change in color of molecular robots [34]. Association ratios of 85–100% were obtained for all the systems corresponding to the output as swarming, which are significantly higher than those for the outputs in which swarming was not realized through logic operations (<5%). Such logical operations of molecular robots based on molecular computation of DNA have not been realized for other swarm robot systems.

### 2.8. Orthogonality in swarming of molecular robots

Orthogonality has been one of the biggest challenges in the operation of molecular robots, particularly with respect to their swarming. We define orthogonality in the context of swarm robotics as a feature that enables swarming or solitary activity of different groups/types of robots without interacting or interfering with each other. Molecular robots making up an orthogonal system are strongly connected to the robots of their own group but still lacks the ability to interact with robots from other groups. Orthogonality is useful in providing groups of robots with enhanced precision in accomplishing multiple tasks simultaneously. The high molecular recognition ability of DNA forms the basis of orthogonal swarming. Information carried by a DNA sequence can be transmitted only to the target molecules without interfering with any other input signals. By making use of such orthogonality, it has been possible to independently control the group formation of molecular robots and swarming even in a complex assembly of different types of robots. For example, two types of DNA input signals were designed, one for a rigid molecular robot and the other one for a flexible molecular robot prepared from microtubules. Mechanical properties of microtubules were modulated by manipulating their polymerization conditions [82]. The rigid and flexible microtubules are different in their path persistence length when driven by kinesins [34]. The rigid and flexible microtubules have been reported also for their variation in morphology when employed in self-assembly. Rigid microtubules produced stiff bundles whereas flexible microtubules produced ring-shaped structures in the self-assembly [83,84]. The bundles and ring-shaped assembled structures exhibited translational and rotational motion, respectively, when they were driven by kinesins. Thus, when DNA were encoded with information that allowed swarming of the rigid and flexible molecular robots, they exhibited concurrent swarming with translational and rotational motion, respectively, without interfering with each other. Thereby, making use of the advantages of DNA a translating swarm, or a rotating swarm, or both the translating and rotating swarm could be operated simultaneously without interfering with each other in an orthogonal fashion [34].

### 2.9. Photo-regulation of swarming of molecular robots

Light was successfully used to drive a nanocar, although coordinated motion of many cars was not accomplished [22]. Photo-regulation of swarming of microtubules has been realized in a reversible and repeatable manner. By incorporating photosensitive azobenzene moiety into DNA strands, the swarm formation by molecular robots and dissociation of swarms has been controlled simply using light (Figure 4). Under ultraviolet light irradiation (λ = 365 nm), the DNA computing element can be transformed to the ‘OFF’ state, and no swarm were formed at this state. When visible light (λ = 480 nm) was applied, the DNA computing element turned ‘ON’ and the formation of swarm begins. By adjusting the physical properties of the molecular robots as described above, it was also possible to control the behavior of the group, such as translation and rotation, by light simultaneously with formation and dissociation of groups.

## 3. Conclusions and future perspectives

**Figure 6.**
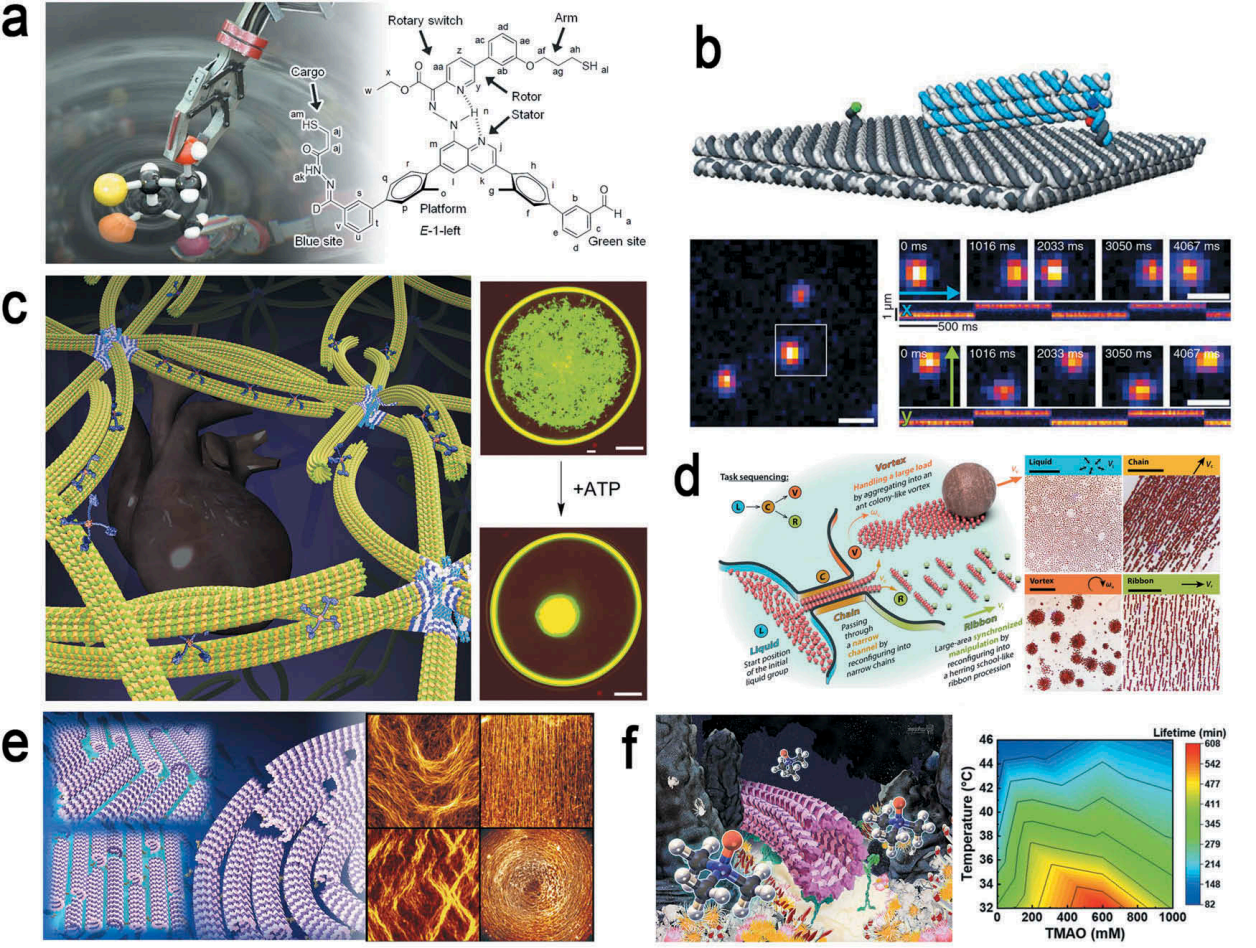
Schematic illustration shows recent advances in the developments of the molecular robots in various forms and their potential applications. (a) Chemical robot arm; Copyright: Prof. David A Leigh; reproduced with permission from [19] and (b) DNA robot arm; reproduced with permission from [29], (c) artificial muscle-like structure fabricated from biomolecular motors; reproduced with permission from [74]. (d) swarming of magnetic microrobots; reproduced with permission from [24], and (e) swarming and pattern formation by robots under mechanical stimulation; reproduced with permission from [76], (f) enhancing the thermal stability of robots using the deep-sea osmolyte trimethylamine N-oxide (TMAO); reproduced with permission from [93].

By overcoming the hurdles related to the size and number of individual molecular robots prepared from biomolecular motors, DNA, and photosensitive molecules, swarming of molecular robots have been executed as an emergent function. The size of robots has been scaled down from centimeters to nanometers, and the number of robots participating in swarming has been successfully increased from one thousand to millions. Further optimization of the molecular robots is necessary for their applications to process, store, and transmit information which are subject to future work (Figure 6). Molecular robots with more complex structures and functions or entirely new frameworks are also being considered in various combinations. For example, apart from the many efforts based on DNA and related nanostructures, there have been reports on the fabrication of peptide-based nanomaterials for artificial systems [85–87]. Being motivated by the 2016 Nobel Prize in Chemistry a great initiative has been undertaken recently for interdisciplinary collaboration to prepare hybrid molecular engine by utilizing synthetic molecular motors created based on supramolecular chemistry, DNA nanotechnology, and biological molecular motors as reported elsewhere [88]. Despite these ongoing progresses, there are several issues to address for the practical applications of molecular robots such as energy efficiency and reusability. From the perspective of sustainable development goals, it would be intriguing to take further initiatives in the future to tackle these challenges related to energy crisis [89]. On the other hand, short lifetime of the robots, particularly of the actuators, due to mechanical aging [90,91] and thermal denaturation pose big drawbacks to the molecular robots [92,93]. To make the molecular robots more sustainable further improvement is necessary to prevent the degradation or functional inactivation of the robots as inspired by using reactive oxygen species-free environment, and osmolytes, etc. [93–96]. In the long run, the molecular robots are expected to greatly contribute to the emergence of a new dimension in chemical synthesis, molecular manufacturing, and artificial intelligence based on fusion of biotechnology, nanotechnology, and informatics.
